# New Method of Inducing Premature Delivery

**Published:** 1854-10

**Authors:** 


*New Method of inducing Premature Delivery.*—Dr. Scanzoni was induced, by observing the active sympathy between the breasts and the other parts of the sexual apparatus, to try to produce premature delivery by irritating the nerves of the mammary glands. The first experiment was made upon a young woman, aged 24, who, two years ago, had been delivered by perforation, in consequence of contraction of the pelvis. In the thirty-second week of utero-gestation, apparatus constructed of caoutchouc, forming sucking-pumps, were put upon the nipples. During three days they were used about seven times, the process going on upon each occasion for two hours. After the third application, the neck of the uterus became shortened; after the sixth, severe labor-pains came on; after the seventh, the child was born.

The only danger likely to ensue from this very simple method of treatment is inflammation of the mammæ; this can be met with proper treatment.

A second case, of similar kind, occurred to the author. A young woman, *enceinte* for the first time, suffered so severely from dyspnœa, connected with organic disease of the chest, that premature delivery was necessary for the perservation of her life. After the third application of the sucking-pumps, an apparently dead child was born; respiration, however, was soon re-established. The author remarks that this case is not quite conclusive, because premature delivery occurs often in connection with severe dyspnœa, independent of other influences.—*Med. Times and Gaz.*, Oct. 1, 1853, from *Verhandl. der Med. Phys. Ges. zu Würtzburg*, 1853.

